# Effect of Storage Temperature and Time on Biogenic Amines in Canned Seafood

**DOI:** 10.3390/foods11182743

**Published:** 2022-09-07

**Authors:** Yinghong Qu, Jingyu Wang, Zhidong Liu, Xichang Wang, Huimin Zhou

**Affiliations:** 1 Shanghai Engineering Research Center of Aquatic Product Processing and Preservation, College of Food Science and Technology, Shanghai Ocean University, Shanghai 200120, China; 2 Key Laboratory of Oceanic and Polar Fisheries, Ministry of Agriculture and Rural Affair, East China Sea Fishery Research Institute, Chinese Academy of Fishery Sciences, Shanghai 200090, China

**Keywords:** biogenic amines, canned seafood, storage time, storage temperature

## Abstract

Biogenic amines in canned seafood are associated with food quality and human health. In this study, a total of nine biogenic amines (histamine (HIS), phenylethylamine (PHE), tyramine (TYM), putrescine (PUT), cadaverine (CAD), tryptamine (TRY), spermine (SPM), spermidine (SPD), and octopamine (OCT)) were used as standards. The biogenic amines of five canned seafood species (canned mud carp, canned sardine, canned mantis shrimp, canned scallop, and canned oyster) were investigated every three months for 12 months at different storage temperatures (4, 10, 25, and 30 °C). The biogenic amine contents were determined by the ultrasound-assisted dispersive solid-phase extraction method combined with reversed-phase high-performance liquid chromatography-photodiode array detection (UADSPE-RPLC-PDA). These results showed a detection rate of 100, 60, and 40% for HIS, PHE, PUT, and TYM; CAD, SPM, and SPD; OCT in all the samples, respectively. The contents of histamine and tyramine exceeded the recommended maximum limits (50 and 100 mg kg^−1^) in the canned mud carp and canned scallop when stored at 30 °C, indicating their potential health risks (*p* < 0.05). This result also indicates that low temperatures could inhibit the BAs content of canned seafood during storage. Overall, storage temperature and time can be used as the primary means to monitor and control the quality and safety of canned seafood.

## 1. Introduction

Canned seafood is recommended due to its good nutritional value, ease of storing at room temperature, and high stability [1]. Canned seafood is generally considered safe, which is achieved through a heat treatment sufficient to kill the vegetative bacteria and bacterial spores and being stored in closed vessels. However, many studies have indicated that the storage environmental factors could significantly affect the quality and safety of canned seafood. Aquatic raw materials are rich in protein; therefore, the protein decomposition of canned aquatic products leads to the formation of biogenic amines (BAs) by microorganism-induced amino acid decarboxylase or the transamination of aldehydes or amino acid transaminases during processing and storage [2]. As such, a low BAs level is acceptable for human health. However, excessive BAs accumulation by the human body could be toxic, resulting in serious public health and food safety concerns [3,4]. The common BAs found in food mainly include HIS, TYM, PUT, and CAD. HIS and TYR pose severe acute effects on human health. PUT and CAD pose low toxicological properties that can act as a precursor of carcinogenic N-nitrosamines under the presence of nitrite [5,6]. The sum of certain BAs are the primary indicators of the quality and safety of aquatic products [6]. The formation of BAs in canned seafood might have significant effects on the nutritional and quality and safety of canned products after yielding, which is mediated by various factors, including the raw materials, microorganisms, and processing and storage conditions, etc. [7,8]. The BAs contents of canned seafood vary to a great extent due to the differences in species, sex, season, stage of maturity, feeding, living environment, and the style of raw materials. The USA Food and Drug Administration (FDA) stipulates that the intervention level of HIS in fish products in the United States is 50 mg kg^−1^ based on the toxicity of HIS at 500 mg kg^−1^ [9]. HIS is the only BA with regulatory limits. Hence, the production and accumulation of excessive BAs content in canned seafood should be limited during storage [10,11]. Thus, the storage temperature and time are of critical importance to controlling BAs contents in canned seafood during storage.

However, the effect of storage conditions (storage temperature range, storage time, etc.) on certain quality and safety characteristics on the quality and safety of canned seafood species is very scarce and unsystematic, especially BAs. Moreover, the profile and content of BAs vary from canned seafood species to species [12]. Although some studies have focused on this aspect, those are limited to a few species, such as tuna, mackerel, sardines, crustaceans, and mollusks [7,8]. This study aimed to investigate the changes in BAs content in various canned seafood species (canned mud carp, canned sardine, canned mantis shrimp, canned scallop, and canned oyster) stored under different conditions (4, 10, 25 and 30 °C) for 12 months and probed into the quality and safety changes during storage.

## 2. Materials and Methods

### 2.1. Samples

Commercially sterilized canned seafood (canned mud carp (*Cirrhinus molitorella*), canned sardine (*Sardinops melanostictus*), canned mantis shrimp (*Portunus trituberculatus*), canned scallop (*Patinopecten yessoensis*), and canned oyster (*Carnis Ostreae*) (*n* = 300)) without damage and rust were purchased from the canned seafood manufacturers (latest production batch) from the 1st to the 7th of August 2020. These are prevalent product in China market.

### 2.2. Determination of Biogenic Amines

A total of nine BAs (PUT, CAD, SPM, SPD, TYR, OCT, PHE, HIS, and TRM) were determined in the canned seafood by the ultrasound-assisted dispersive solid-phase extraction method combined with reversed-phase high-performance liquid chromatography-photodiode array detection (RP-HPLC-PDA) (Waters 2695 Separations Module, Waters, Milford, CT, USA) [13]. About 5 g of aliquot was weighed and placed in a 50 mL centrifuge tube. Later, 20 mL of TCA (5%, *w*/*v*) was added, and the mixtures were homogenized for 3 min (Ouhor, Shanghai, China), followed by ultrasonication for 30 min (KO5200E ultrasonic bath, Kun Shan Ultrasonic Instruments Co., Shanghai, China), and finally centrifuged at 5000 rpm for 10 min. The supernatant was transferred to a 50 mL brown volumetric flask, and the volume was fixed to the scale with 5% trichloroacetic acid. Afterward, 10 mL of n-hexane was added to the above sample extraction solution (10 mL) for complete fat removal, and the ultrasonic treatment was repeated for 10 min. After static stratification, the upper organic layer was discarded. About 5 mL of the degreased solution was transferred to a 50 mL centrifuge tube. The pH was alkalized up to 12 by adding NaOH (2 mol L^−1^) (Mettler Toledo, Greifensee, Switzerland). About 5 mL of n-butanol/chloroform (1/1) mixed solution was added to purify the extracted solution. After being ultrasonicated for 10 min and centrifuged at 5000 rpm for 5 min, the upper organic layer was adjusted to 10 mL by adding the mixed solution of n-butanol/chloroform (1/1). Finally, 5 mL of the above purified extracted solution was taken into the test tube and blown to dryness by nitrogen in a 40 °C water bath. After being added to 1 mL of hydrochloric acid (0.1 mol L^−1^) and ultrasonicated for 5 min, the extract could be used for derivatization and determination according to the method reported by Ishimaru, et al. and Gong, et al., respectively [14,15,16]. All standards/compounds were separated and identified by their retention times. The reagents were procured from Sigma-Aldrich (Goettingen, Germany) (amines and dansysl chloride) or Sigma-Aldrich (St. Louis, MO, USA) (acetonitrile, acetone, n-butanol, and n-hexane). The results are expressed in mg kg^−1^ wet weight.

The chromatograms containing BAs in the standard solutions showed a good peak resolution, with selectivity, sharpness, and symmetry. The correlation coefficients of the linear regression lines were better than 0.999 for all compounds. The LODs and LOQs of all BAs ranged between 0.08 and 0.25 mg kg^−1^ and 0.27 to 0.83 mg kg^−1^, respectively. The precision, repeatability, reproducibility, recovery, and accuracy values of this method were assessed according to the previously reported method [9]. These results were consistent with the previous study results [9,17], demonstrating the feasibility of this method to detect BAs content in canned seafood.

### 2.3. Storage Experiments

Canned seafood (canned mud carp, canned sardine, canned mantis shrimp, canned scallop, and canned oyster, *n* = 300) was used as the samples for the storage experiment. These samples were stored at 4, 10, 25, and 30 °C and the BAs content was determined every three months during the storage for 12 months, respectively. Different canned seafood species were randomly collected and mixed with three samples from each species at the scheduling points to ensure the uniformity and representativeness of the samples.

### 2.4. Index Value

The synergism of various BAs induces toxicity in canned seafood, therefore, evaluating the biogenic amine index (BAI) is necessary to determine its quality. The interrelationship of HIS, PUT, CAD, SPD, and SPM was used to formulate a chemical index of decomposition for canned seafood [17]. This index was consistent with the sensory evaluation scores, confirming its adequacy for the quality evaluation of seafood products [18,19]. The relationship between the 5 amines was analyzed according to the 3 sensory quality classes, with the following generated formula:

Chemical Index = (HIS + PUT + CAD)/(1 + SPM + SPD)(1)

where, PUT, CAD, HIS, SPD, and SPM are the contents (mg kg^−1^) of putrescine, cadaverine, histamine, methylamine spermidine, and spermine, respectively. A chemical index of BAs within 0–1 indicated the good quality of canned seafood, while a range higher than 10 would correspond to an unacceptable quality [20].

The chemical index level was not consistent for different species; the levels were even different for two differently packed products of the same type. Therefore, the chemical index should be studied along with other quality indicators. The amine index proposed by Duflos, et al., (1999) [21] was used to assess the spoilage:

Amine Index = (HIS + PUT + CAD)/(HIS + PUT + CAD + TRM + TYR + PHE + SPD + SPM) × 100(2)

where, PUT, CAD, HIS, TRM, TYR, PHE, SPD, and SPM are the contents (mg kg^−1^) of putrescine, cadaverine, histamine, tryptamine, tyramine, phenylethylamine, spermidine, and spermine, respectively. According to the defined reference limits, the amine index values of below 25 indicate good quality, while the index values higher than 66 correspond to inedibility.

### 2.5. Statistical Analysis

Statistical analysis was performed using Origin (OriginLab Inc., Northampton, MA, USA) and SPSS 26.0 software (SPSS Inc., Chicago, IL, USA). Data were analyzed for the degree of variation by calculating the mean of at least three determinations and standard deviations (SDs) of the results. The significance of differences was evaluated using a one way analysis of variance (one-way ANOVA). A *p* value of less than 0.05 was considered statistically significant.

## 3. Results

### 3.1. Changes of BAs in Canned Mud Carp during Storage

The total BAs content in canned mud carp stored at 4, 10, 25, and 30 °C significantly increased (*p* < 0.05) with prolonged storage time. A total of even BAs (TRM, PHE, PUT, HIS, TYR, SPD, and SPM) was detected in caned mud carp during storage, without the occurrence of CAD and OCT (Table 1). After being stored at 4, 10, 25, and 30 °C, the total BAs in canned mud carp increased from the initial values of 7.02 mg kg^−1^ to 40.56, 57.25, 79.64, 45.00, and 122.71 mg kg^−1^, respectively. The sum of all seven amines was <300 mg kg^−1^ in all samples (maximum value: 122.71 mg kg^−1^). The higher the storage temperature, the higher the total BAs in caned mud carp. This obtained value was much lower than the results reported by Kim, et al. (2009) [22]. When canned mud carp was stored at 4, 10, 25, and 30 °C for 12 months, the HIS content reached 15.32, 23.74, 27.36, and 52.81 mg kg^−1^, respectively. Thus, based on the limit levels of HIS set by the USA FDA (50 mg kg^−1^), canned mud carp is unsuitable for storage at 30 °C for 12 months. When canned mud carp was stored at 4, 10, 25, and 30 °C for 12 months, the SPM level reached 5.32, 8.74, 15.59, and 17.47 mg kg^−1^, respectively. TRM and PUT levels showed a significantly increasing trend when stored at 4 and 10 °C, while SPD showed a significantly increasing trend at 25 and 30 °C (*p* < 0.05). The SPM level changed the most when canned mud carp was stored at 25 °C. This might have happened as the pH of canned seafood content increased with the increased storage temperature. Previous studies have also reported correlations between pH and BAs formation in seafood [23].

### 3.2. Changes of BAs in Canned Sardine during Storage

Fish is widely consumed among protein-rich foods [24]. The total BAs in the canned sardine significantly increased with the storage temperature increasing for 12 months (*p* < 0.05). A total of eight BAs (TRM, PHE, PUT, HIS, OCT, TYR, SPD, and SPM) were detected in the canned sardine during storage without the occurrence of CAD, HIS, and OCT (Table 2). After storage at 4, 10, 25, and 30 °C, the total BAs in canned sardine increased from the initial values of 13.29 mg kg^−1^ to 71.21, 91.61, 146.00, and 158.52 mg kg^−1^, respectively. Meanwhile, the content increased at a faster rate at 30 than at 4 °C. The sum of all eight amines was <300 mg kg^−1^ in all samples (maximum value: 158.52 mg kg^−1^). HIS significantly increased in all samples during storage and decreased slowly after 6 months of storage. Similarly, SPD and SPM significantly increased (*p* < 0.05) when stored at 4 °C; TRM and OCT significantly increased (*p* < 0.05) when stored at 10 and 25 °C; PHE, SPD, and OCT significantly increased (*p* < 0.05) when stored at 30 °C. However, there was no significant difference in TYR and CAD (*p* > 0.05) when stored at 4, 10, and 30 °C. Notably, CAD was detected, which significantly increased (*p* < 0.05) when stored at 25 °C. This might have happened because CAD could not be decomposed at room temperature. These eight BAs showed an increasing tendency with increased storage temperature (Table 2), which was consistent with the results of Gómez-Limia, et al. (2020) [7]. Bilgin and Gençcelep also detected TYM in canned tuna, chunk canned tuna, marinated anchovies, canned mackerel, and canned sardines at levels ranging between ND and 48.63 mg kg^−1^ [23]. Gómez-Limia, et al. (2020) reported that the hydrolysis reactions and the interactions of the components in canned seafood continued when stored at room temperature [7].

### 3.3. Changes of BAs in Canned Mantis Shrimp during Storage

The total BAs in the canned mantis shrimp significantly increased with the increase in temperatures for 12 months (*p* < 0.05). A total of seven BAs (TRM, PHE, PUT, CAD, HIS, OCT, and TYR) were detected in canned mantis shrimp during storage (Table 3). No SPD or SPM was detected during storage, which was different from canned mud carp, canned sardine, and canned oyster due to the differences in species. The total BAs content in canned sardine increased from the initial values of 8.59 mg kg^−1^ for 12 months to 50.21, 76.42, 123.57, and 145.14 mg kg^−1^, respectively, when stored at 4, 10, 25, and 30 °C. The sum of all seven amines was <300 mg kg^−1^ in all samples (maximum value: 145.14 mg kg^−1^). Among all BAs, HIS showed a significant increase (*p* < 0.05) during storage and reached 12.46, 22.47, 33.74, and 42.18 mg kg^−1^, respectively. The higher the storage temperature, the higher the HIS content. It was found that PUT, CAD, OCT, and TYR significantly increased (*p* < 0.05) when stored at 4 °C; PHE, PUT, CAD, and TYR significantly increased (*p* < 0.05) when stored at 10 °C; with further increases in temperature, PHE, PUT, CAD, OCT, and TYR significantly increased (*p* < 0.05) at 25 and 30 °C. Therefore, PHE, PUT, CAD, HIS, and OCT could be used as the characteristic BAI of canned mantis shrimp during storage. These seven BAs showed an increasing tendency with increased storage temperature (Table 3). Zhai et al. reported that the total BAs content in different canned fish products ranged from 1.94 to 112.54 mg kg^−1^, with a mean value of 46.43 mg kg^−1^ [25], which was consistent with the present study result.

### 3.4. Changes of BAs in Canned Scallop during Storage

As summarized in Table 4, only five BAs (TRM, PHE, PUT, CAD, and HIS) were detected in canned scallops during storage, which was consistent with canned mantis shrimp. Although the total BAs content in canned scallop was much lower than those in canned sardine and canned scallop, they also showed a significantly increasing trend with increasing storage temperature for 12 months (*p* < 0.05). The total BA in canned sardine increased to 33.29, 59.94, 76.68, and 90.79 mg kg^−1^, respectively, when stored at 4, 10, 25, and 30 °C for 12 months. The sum of all five amines was <300 mg kg^−1^ in all samples (maximum value: 90.79 mg kg^−1^). HIS showed a significant increase (*p* < 0.05) in all the BAs during storage and reached 23.05, 39.86, 49.57, and 52.39 mg kg^−1^, respectively, which was more than the limit standard of FDA (50 mg kg^−1^). TRM, PUT, PHE, and CAD significantly increased (*p* < 0.05) when stored at 10, 25, and 30 °C, while OCT, TYR, SPD, and SPM were not detected. PHE showed no significant difference when stored at 4 °C (*p* > 0.05), maintaining an overall growth trend [15]. These five BAs showed an increasing tendency with increased storage temperature (Table 4). Nevertheless, the values reported for total BAs content in samples are low, well below FDA-recommended levels at 4, 10, and 25 °C [19].

### 3.5. Changes of BAs in Canned Oyster during Storage

The total BAs in canned oyster significantly increased with the increase of storage temperature for 12 months (*p* < 0.05), showing a faster rate at 30 than at 4 °C. As summarized in Table S1, a total of seven BAs (TRM, PHE, PUT, CAD, HIS, SPD, and SPM) were detected in the canned oyster during storage. TRM, PUT, CAD, HIS, OCT, TYR, and SPM were detected in the initial phase. The total BAs content in canned oyster increased from the initial value of 2.54 mg kg^−1^ to 52.11, 77.92, 100.97, and 122.62 mg kg^−1^, respectively, when stored at 4, 10, 25, and 30 °C. The sum of all seven amines was <300 mg kg^−1^ in all samples (maximum value: 122.62 mg kg^−1^). HIS significantly increased in all BAs during storage and decreased slowly after 3 months of storage. TRM and SPM significantly increased (*p* < 0.05) when stored at 4 °C; TRM and CAD significantly increased (*p* < 0.05) when stored at 10 °C; TRM, SPD, and CAD significantly increased (*p* < 0.05) when stored at 25 °C; TRM, SPD, PUT, and CAD significantly increased (*p* < 0.05) when stored at 30 °C. However, TYR and OCT were not detected during storage at 4, 10, 25, and 30 °C for 12 months. Notably, CAD was detected, which significantly increased (*p* < 0.05) when stored at 10 °C. This might have happened because CAD in canned oyster could not be decomposed at 10 °C. These seven BAs (TRM, PHE, PUT, CAD, HIS, SPD, and SPM) showed an increasing tendency with increased storage temperature. The total BAs content increased during storage for 12 months, which was consistent with Gómez-Limia, et al. (2020) [7].

### 3.6. Formulas for BAs Content in Canned Seafood under Different Storage Conditions

The Chemical Index and Amine Index of all the samples were determined according to Equations (1) and (2), and the results are summarized in Table S2. It can be seen that the initial Chemical Index and Amine Index of canned fish were less than 2.5 and 60, respectively, indicating good quality. Different varieties have different quality standards due to the differences in raw materials, processing technology, and storage environment. Therefore, the Chemical Index and Amine Index range of less than 55/55 and 75/90 might correspond to good quality of canned shrimp and shellfish, respectively, while the range higher than 30 and 68 might indicate the quality change of the samples. If one of the two indicators meets the limited standards, the sample would be considered poor quality. Therefore, more studies on the indicative correlation of BAs are demanded to gain insights into the quality characteristics of canned seafood.

Moreover, the sensory properties stored at 4 and 10 °C were better than those stored at 25 and 30 °C, indicating that low storage temperatures could better obtain the quality of canned seafood. With prolonged storage, the Chemical Index and Amine Index of canned mud carp, canned sardine, canned scallop, and canned oyster exceed the human consumption limits recommended by FAO/WHO [26], indicating that different degrees of deterioration negatively affect the quality of these samples. Nevertheless, these samples are still safe for human consumption due to BAs’ limited content. The canned mantis shrimp and canned scallop had a more obvious response to storage time and temperature, showing significant quality changes. The canned mantis shrimp and canned scallop samples decomposed when stored at 25 and 30 °C for more than 6 months. There was no significant change in the quality of canned oysters when stored at any temperature for one year. Thus, temperature control could effectively inhibit BAs’ formation in canned seafood during storage. The changes in BAs’ contents in the canned seafood were relatively low and avoided possible harmful effects under low temperature storage. Therefore, further studies on the storage conditions of canned seafood could guarantee the quality and safety of canned seafood. Additionally, different species of aquatic canned products could affect BAs’ formation. Canned scallop and canned oyster produced more HIS than canned mud carp, canned sardine, and canned mantis shrimp at 4 °C. The correlation between BAs and other quality characteristics should be studied further to construct the quality and safety prediction model and reveal the changing tendency of BAs content in canned seafood during storage.

## 4. Discussion

BAs levels of canned seafood depend on the intrinsic food factors, such as pH, *a*_w_ values, and nutrients, and the extrinsic factors, including storage time, temperature, etc., [6]. Shalaby (1996) reported that the BAs concentrations differ not only between different food varieties but also within the same variety [27]. With storage time prolonged, BAs will accumulate. Especially CAD and PUT in BAs increased with the increased temperature [28]; however, storage at low temperatures might induce PUT accumulation [29]. Thus, higher BAs contents were detected in the canned seafood with higher storage temperatures. Pinho et al. (2001) reported a higher BAs content at a storage temperature of 21 °C than that stored at 4 °C [30]. Kim et al. (2002) demonstrated that the optimum temperature for HIS production in fish product was 25 °C [31]. These results were consistent with the present study results. This indicates that adjusting the storage environment factors (storage time and temperature, etc.) during storage is the primary means of controlling the quality and safety of canned seafood after manufacturing [32]. Other studies have reported the effect of storage temperature on HIS formation in fish products, such as tuna and anchovies [33,34]. Moreover, low HIS and TYR contents have been reported in crustaceans such as shrimp [35]. These results were consistent with previous reports [36].

FAO/WHO stipulates a level of 200mg kg^−1^ for fish and fishery products, and EFSA stipulates 400 mg kg^−1^ for fishery products, respectively, [19,36,37]. According to a previous study, HIS and TYR exert severe acute effects on human health, while, PUT and CAD have low toxicological properties [6]. According to EFSA, TYR values of 600 mg kg^−1^ or more are good for healthy individuals [36]. The TYR contents in all the samples were less than 600 mg kg^−1^ (Table 1, Table 2, Table 3 and Table 4), which was consistent with Prester et al., who reported a dietary value of up to 800 mg kg^−1^ of TYR to be acceptable [35]. Other studies have also reported that the acute toxicity levels of TYR and CAD are significantly greater than 2000 mg kg^−1^ and the oral toxicity level of PUT is 2000 mg kg^−1^ [38]. It is known that TYR has a stronger and more rapid cytotoxic effect than HIS [39], while the bioactivities of PUT and CAD are less potent. Furthermore, PUT and CAD significantly increased with increased storage times, resulting in strong unpleasant decaying odors at very low concentrations [40]. These results were consistent with previous studies that canned seafood should be considered as a low-risk product based on the BAs contents [36]. Poveda (2019) reported that PUT is either converted to SPD or SPM or is catabolized by succinate [41]. Gezginc et al. (2013) found that arginine serves as a precursor of PUT, which can also be converted to SPD [42,43]. Furthermore, for the BAs used as the indicative of food quality, they are mostly produced at the end of shelf-life of food. This index was also used to indicate the evaluation and comparison of BAs contents in food [44]. Therefore, the storage temperature and time of canned seafood for different varieties should be studied more comprehensively to minimize the BAs content and obtain good quality.

## 5. Conclusions

The present study evaluated the BAs content in canned seafood during storage at different temperatures and times. HIS exhibited the most significant changes under different storage conditions. HIS content in the canned sardine and canned mantis shrimp increased to 50 mg kg^−1^ when stored at 25 and 30 °C for one year, while it increased to more than 50 mg kg^−1^ in the canned mantis shrimp and canned scallop when stored at 30 °C. TRM, PUT, and HIS could be used as the characteristic BAs of canned seafood during storage. These findings provide deeper insights into the influence of storage time and temperature on BAs level, which can be used to improve the quality and safety of canned seafood.

## Figures and Tables

**Table 1:** Changes of BAs in canned mud carp during storage at different temperatures.

BA Contents/(mg kg^−1^)	Temperature (°C)	Time (Month)				
		0	3	6	9	12
TRM	4	1.57 ± 0.08 ^c^	3.84 ± 0.56 ^A,b^	3.99 ± 0.82 ^A,b^	5.17 ± 0.52 ^A,a^	5.38 ± 0.36 ^B,a^
	10	1.57 ± 0.08 ^c^	1.43 ± 0.32 ^C,c^	1.69 ± 0.04 ^B,c^	2.57 ± 0.35 ^C,b^	5.82 ± 0.28 ^B,a^
	25	1.57 ± 0.08 ^e^	2.58 ± 0.26 ^B,d^	3.66 ± 0.31 ^A,c^	4.96 ± 0.27 ^A,b^	6.73 ± 0.23 ^A,a^
	30	1.57 ± 0.08 ^d^	3.48 ± 0.24 ^A,b^	3.45 ± 0.37 ^A,b^	3.90 ± 0.42 ^B,b^	6.92 ± 0.77 ^A,a^
PHE	4	1.02 ± 0.09 ^b^	1.33 ± 0.17 ^B,b^	1.38 ± 0.04 ^C,b^	2.43 ± 0.77 ^B,a^	2.91 ± 0.09 ^C,a^
	10	1.02 ± 0.09 ^c^	1.94 ± 0.93 ^B,bc^	2.54 ± 0.36 ^B,b^	3.83 ± 0.56 ^B,a^	4.63 ± 0.12 ^B,a^
	25	1.02 ± 0.09 ^d^	1.98 ± 0.17 ^B,c^	2.76 ± 0.37 ^B,c^	3.89 ± 0.34 ^B,b^	4.99 ± 0.81 ^B,a^
	30	1.02 ± 0.09 ^e^	3.02 ± 0.41 ^A,d^	5.13 ± 0.79 ^A,c^	11.86 ± 1.37 ^A,b^	13.48 ± 1.02 ^A,a^
PUT	4	1.31 ± 0.03 ^c^	1.48 ± 0.98 ^c^	2.13 ± 0.04 ^C,c^	5.02 ± 0.47 ^B,b^	6.28 ± 0.93 ^B,a^
	10	1.31 ± 0.03 ^d^	1.64 ± 0.05 ^d^	3.37 ± 0.83 ^B,c^	5.18 ± 0.30 ^B,b^	6.99 ± 0.61 ^B,a^
	25	1.31 ± 0.03 ^d^	2.30 ± 0.46 ^cd^	2.58 ± 0.37 ^BC,c^	5.39 ± 0.92 ^B,b^	7.12 ± 0.91 ^B,a^
	30	1.31 ± 0.03 ^e^	2.39 ± 0.63 ^d^	4.29 ± 0.21 ^A,c^	7.03 ± 0.29 ^A,b^	9.74 ± 1.05 ^A,a^
HIS	4	ND ^d^	3.75 ± 0.34 ^C,c^	10.96 ± 1.99 ^B,b^	12.38 ± 1.65 ^C,b^	15.32 ± 2.05 ^C,a^
	10	ND ^d^	2.43 ± 0.49 ^D,d^	15.03 ± 1.42 ^AB,c^	18.45 ± 2.42 ^C,b^	23.74 ± 1.28 ^B,a^
	25	ND ^c^	15.78 ± 0.29 ^A,b^	17.47 ± 1.09 ^A,b^	26.02 ± 3.09 ^B,a^	27.36 ± 2.57 ^B,a^
	30	ND ^e^	8.39 ± 0.12 ^B,d^	20.58 ± 5.99 ^A,c^	38.20 ± 6.20 ^A,b^	52.81 ± 4.02 ^A,a^
TYR	4	0.92 ± 0.09 ^bc^	0.47 ± 0.05 ^C,c^	0.36 ± 0.08 ^C,d^	1.16 ± 0.63 ^C,b^	2.21 ± 0.13 ^C,a^
	10	0.92 ± 0.09 ^d^	1.23 ± 0.40 ^B,cd^	1.58 ± 0.32 ^B,c^	2.48 ± 0.29 ^BC,b^	3.10 ± 0.22 ^C,a^
	25	0.92 ± 0.09 ^d^	1.35 ± 0.49 ^B,cd^	2.30 ± 0.63 ^B,c^	6.43 ± 1.02 ^A,a^	6.91 ± 0.37 ^B,a^
	30	0.92 ± 0.09 ^d^	2.04 ± 0.12 ^A,cd^	3.54 ± 0.88 ^A,b^	3.06 ± 0.93 ^B,bc^	8.31 ± 0.95 ^A,a^
SPD	4	1.51 ± 0.07 ^d^	1.66 ± 0.14 ^A,d^	2.38 ± 0.08 ^B,c^	2.82 ± 0.21 ^B,b^	3.12 ± 0.17 ^C,a^
	10	1.51 ± 0.07 ^c^	1.58 ± 0.09 ^A,c^	1.85 ± 0.12 ^B,b^	1.94 ± 0.12 ^B,b^	4.28 ± 0.24 ^C,a^
	25	1.51 ± 0.07 ^d^	2.07 ± 0.18 ^A,d^	3.54 ± 0.32 ^A,c^	8.09 ± 1.52 ^A,b^	10.75 ± 0.32 ^B,a^
	30	1.51 ± 0.07 ^d^	0.93 ± 0.48 ^B,d^	4.05 ± 0.51 ^A,c^	9.21 ± 1.62 ^A,b^	13.98 ± 1.61 ^A,a^
SPM	4	0.69 ± 0.48 ^c^	0.75 ± 0.51 ^c^	2.74 ± 0.93 ^C,b^	4.35 ± 0.61 ^B,a^	5.32 ± 0.39 ^C,a^
	10	0.69 ± 0.48 ^c^	2.39 ± 1.93 ^c^	5.85 ± 0.23 ^B,b^	8.39 ± 2.01 ^AB,a^	8.74 ± 0.63 ^B,a^
	25	0.69 ± 0.48 ^d^	1.59 ± 0.20 ^d^	5.06 ± 0.09 ^B,c^	12.49 ± 3.03 ^A,b^	15.59 ± 0.37 ^A,a^
	30	0.69 ± 0.48 ^c^	1.49 ± 0.18 ^c^	8.08 ± 0.37 ^A,b^	9.30 ± 3.21 ^A,b^	17.47 ± 2.19 ^A,a^

ND: not detected. Within each column and for each storage time of each amine, different capital letters (A–D) indicate significant differences (*p* < 0.05); within each row and for each storage temperature, different lowercase letters (a–e) indicate significant differences (*p* < 0.05). The absence of a letter indicates that no significant differences were found (*p* > 0.05).

**Table 2:** Changes of BAs in canned sardine during storage at different temperatures.

BA Contents/(mg kg^−1^)	Temperature (°C)	Time (Month)				
		0	3	6	9	12
TRM	4	5.14 ± 0.34 ^b^	5.60 ± 0.64 ^ab^	6.48 ± 1.37 ^A,ab^	7.03 ± 1.09 ^C,a^	7.21 ± 0.96 ^C,a^
	10	5.14 ± 0.34 ^c^	5.58 ± 0.66 ^c^	5.90 ± 0.79 ^AB,c^	9.45 ± 1.07 ^B,b^	12.49 ± 0.18 ^B,a^
	25	5.14 ± 0.34 ^d^	5.26 ± 0.45 ^d^	6.23 ± 1.18 ^AB,c^	13.06 ± 0.19 ^A,b^	16.48 ± 0.85 ^A,a^
	30	5.14 ± 0.34 ^c^	5.14 ± 1.04 ^c^	5.87 ± 0.45 ^B,c^	8.39 ± 0.34 ^BC,b^	15.68 ± 1.45 ^A,a^
PHE	4	1.30 ± 0.09 ^d^	2.04 ± 0.28 ^B,c^	2.95 ± 0.12 ^D,bc^	3.48 ± 0.77 ^C,b^	5.23 ± 0.83 ^C,a^
	10	1.30 ± 0.09 ^c^	3.78 ± 0.15 ^A,b^	4.32 ± 0.49 ^C,b^	4.59 ± 1.21 ^C,ab^	5.68 ± 0.38 ^C,a^
	25	1.30 ± 0.09 ^d^	3.05 ± 0.05 ^A,c^	5.88 ± 0.28 ^B,b^	8.93 ± 1.24 ^B,a^	8.98 ± 0.97 ^B,a^
	30	1.30 ± 0.09 ^e^	3.69 ± 0.89 ^A,d^	7.46 ± 0.84 ^A,c^	12.73 ± 1.92 ^A,b^	18.79 ± 0.91 ^A,a^
PUT	4	1.82 ± 0.05 ^b^	2.45 ± 0.51 ^AB,b^	2.84 ± 0.38 ^B,b^	8.24 ± 0.95 ^A,a^	8.32 ± 0.59 ^B,a^
	10	1.82 ± 0.05 ^e^	2.89 ± 0.37 ^A,d^	3.77 ± 0.39 ^AB,c^	8.48 ± 0.12 ^A,b^	9.24 ± 0.74 ^B,a^
	25	1.82 ± 0.05 ^d^	1.83 ± 0.26 ^B,d^	3.50 ± 0.28 ^AB,c^	7.72 ± 0.31 ^AB,b^	9.34 ± 0.92 ^B,a^
	30	1.82 ± 0.05 ^d^	3.09 ± 0.87 ^A,cd^	4.28 ± 0.86 ^A,c^	6.32 ± 1.12 ^B,b^	12.27 ± 1.43 ^A,a^
HIS	4	ND ^c^	6.94 ± 0.74 ^C,b^	14.96 ± 1.90 ^B,a^	16.82 ± 2.15 ^C,a^	17.38 ± 2.01 ^D,a^
	10	ND ^d^	13.54 ± 1.53 ^B,c^	18.58 ± 1.96 ^B,b^	20.14 ± 1.69 ^C,b^	25.39 ± 2.96 ^C,a^
	25	ND ^c^	ND ^D,c^	29.34 ± 1.53 ^A,b^	35.87 ± 3.40 ^B,a^	37.86 ± 4.83 ^B,a^
	30	ND ^c^	28.48 ± 3.19 ^A,b^	31.32 ± 3.50 ^A,b^	42.02 ± 3.03 ^A,a^	46.29 ± 3.01 ^A,a^
TYR	4	0.95 ± 0.40 ^b^	1.02 ± 0.04 ^B,b^	0.93 ± 0.28 ^C,b^	1.88 ± 0.13 ^C,a^	2.40 ± 0.41 ^D,a^
	10	0.95 ± 0.40 ^c^	1.33 ± 0.48 ^B,c^	1.43 ± 0.53 ^BC,c^	4.03 ± 0.21 ^B,b^	5.23 ± 0.86 ^C,a^
	25	0.95 ± 0.40 ^d^	1.97 ± 0.25 ^A,c^	1.99 ± 0.20 ^B,c^	3.24 ± 0.39 ^B,b^	6.38 ± 0.41 ^B,a^
	30	0.95 ± 0.40 ^d^	1.05 ± 0.09 ^B,d^	3.11 ± 0.26 ^A,c^	7.62 ± 1.22 ^A,b^	9.29 ± 0.46 ^A,a^
SPD	4	1.94 ± 0.17 ^c^	3.05 ± 0.29 ^B,bc^	4.55 ± 0.79 ^D,b^	8.99 ± 1.28 ^B,a^	10.42 ± 1.06 ^B,a^
	10	1.94 ± 0.17 ^d^	2.41 ± 0.23 ^B,d^	6.30 ± 0.21 ^C,c^	8.32 ± 0.92 ^B,b^	11.52 ± 2.21 ^B,a^
	25	1.94 ± 0.17 ^c^	2.52 ± 0.35 ^B,c^	9.02 ± 0.88 ^B,b^	9.95 ± 1.40 ^B,b^	12.75 ± 1.37 ^B,a^
	30	1.94 ± 0.17 ^d^	6.03 ± 1.92 ^A,c^	14.35 ± 1.16 ^A,b^	19.01 ± 0.31 ^A,a^	21.38 ± 2.22 ^A,a^
SPM	4	2.14 ± 0.26 ^d^	2.99 ± 0.28 ^C,d^	7.24 ± 0.95 ^AB,c^	12.49 ± 0.47 ^A,b^	13.86 ± 0.77 ^A,a^
	10	2.14 ± 0.26 ^d^	5.02 ± 0.38 ^A,c^	5.94 ± 0.85 ^B,bc^	7.92 ± 0.37 ^C,b^	10.58 ± 2.57 ^B,a^
	25	2.14 ± 0.26 ^d^	3.83 ± 0.57 ^B,c^	6.26 ± 0.24 ^B,b^	8.86 ± 0.62 ^BC,a^	9.56 ± 1.35 ^B,a^
	30	2.14 ± 0.26 ^d^	5.04 ± 0.42 ^A,c^	9.06 ± 1.94 ^A,b^	10.93 ± 2.01 ^AB,b^	14.45 ± 1.47 ^A,a^

ND: not detected. Within each column and for each storage time of each amine, different capital letters (A–D) indicate significant differences (*p* < 0.05); within each row and for each storage temperature, different lowercase letters (a–e) indicate significant differences (*p* < 0.05). The absence of a letter indicates that no significant differences were found (*p* > 0.05).

**Table 3:** Changes of BAs in canned mantis shrimp during storage at different temperatures.

BA Contents/(mg kg^−1^)	Temperature (°C)	Time (Month)				
		0	3	6	9	12
TRM	4	ND ^d^	ND ^d^	1.03 ± 0.08 ^B,c^	1.48 ± 0.39 ^C,b^	2.31 ± 0.21 ^C,a^
	10	ND ^d^	ND ^d^	0.56 ± 0.07 ^B,c^	1.99 ± 0.53 ^BC,b^	3.28 ± 0.17 ^B,a^
	25	ND ^c^	ND ^c^	2.75 ± 0.83 ^A,b^	2.92 ± 0.28 ^B,b^	3.75 ± 0.36 ^B,a^
	30	ND ^c^	ND ^c^	2.58 ± 0.49 ^A,b^	4.39 ± 0.62 ^A,a^	4.81 ± 0.75 ^A,a^
PHE	4	1.84 ± 0.16 ^c^	2.27 ± 0.63 ^C,b^	2.26 ± 0.09 ^C,b^	2.50 ± 0.24 ^C,b^	3.48 ± 0.28 ^D,a^
	10	1.84 ± 0.16 ^c^	4.77 ± 0.85 ^B,b^	4.85 ± 0.37 ^B,b^	7.05 ± 0.99 ^B,a^	7.54 ± 0.51 ^C,a^
	25	1.84 ± 0.16 ^e^	6.11 ± 0.84 ^AB,c^	3.26 ± 0.49 ^C,d^	9.05 ± 0.36 ^A,b^	12.48 ± 1.39 ^B,a^
	30	1.84 ± 0.16 ^d^	6.59 ± 0.82 ^A,c^	6.97 ± 1.38 ^A,c^	9.33 ± 1.71 ^A,b^	14.38 ± 1.13 ^A,a^
PUT	4	2.24 ± 0.07 ^c^	3.95 ± 0.55 ^b^	4.75 ± 0.83 ^C,b^	8.39 ± 0.87 ^C,a^	8.82 ± 0.53 ^C,a^
	10	2.24 ± 0.07 ^b^	3.42 ± 0.37 ^b^	6.40 ± 0.29 ^B,a^	7.82 ± 1.04 ^C,a^	8.72 ± 1.38 ^C,a^
	25	2.24 ± 0.07 ^d^	3.22 ± 0.06 ^d^	8.77 ± 0.59 ^B,c^	10.92 ± 0.89 ^B,b^	20.47 ± 1.21 ^B,a^
	30	2.24 ± 0.07 ^c^	3.62 ± 1.02 ^c^	16.57 ± 3.03 ^A,b^	17.10 ± 2.27 ^A,b^	25.38 ± 1.79 ^A,a^
CAD	4	3.37 ± 0.35 ^d^	4.01 ± 0.19 ^cd^	4.75 ± 0.27 ^D,c^	5.87 ± 0.04 ^C,b^	7.29 ± 1.18 ^C,a^
	10	3.37 ± 0.35 ^d^	4.40 ± 0.59 ^d^	8.54 ± 1.43 ^C,c^	15.49 ± 3.82 ^AB,b^	19.74 ± 2.81 ^B,a^
	25	3.37 ± 0.35 ^c^	3.83 ± 0.25 ^c^	11.82 ± 1.33 ^B,b^	12.43 ± 2.01 ^B,b^	19.31 ± 1.37 ^B,a^
	30	3.37 ± 0.35 ^d^	4.02 ± 0.15 ^d^	15.12 ± 2.85 ^A,c^	18.84 ± 2.57 ^A,b^	28.37 ± 2.15 ^A,a^
HIS	4	ND ^d^	1.31 ± 0.07 ^C,d^	8.05 ± 1.01 ^C,c^	10.84 ± 1.29 ^D,b^	12.46 ± 0.82 ^D,a^
	10	ND ^d^	10.19 ± 0.93 ^A,c^	13.08 ± 3.97 ^B,c^	18.23 ± 1.01 ^C,b^	22.47 ± 1.82 ^C,a^
	25	ND ^d^	10.94 ± 1.07 ^A,c^	13.10 ± 1.81 ^B,c^	28.65 ± 2.98 ^B,b^	33.74 ± 2.39 ^B,a^
	30	ND ^c^	4.58 ± 0.13 ^B,c^	22.94 ± 3.22 ^A,b^	37.10 ± 4.89 ^A,a^	42.18 ± 2.57 ^A,a^
OCT	4	ND ^c^	ND ^B,c^	4.99 ± 0.95 ^B,b^	5.03 ± 1.92 ^BC,b^	8.47 ± 0.41 ^C,a^
	10	ND ^d^	ND ^B,d^	2.55 ± 0.59 ^C,c^	3.90 ± 0.21 ^C,b^	9.95 ± 0.18 ^C,a^
	25	ND ^d^	1.44 ± 0.28 ^A,d^	4.56 ± 0.37 ^B,c^	8.54 ± 1.93 ^B,b^	13.56 ± 0.69 ^B,a^
	30	ND ^d^	ND ^B,d^	8.11 ± 1.05 ^A,c^	14.03 ± 2.69 ^A,b^	19.47 ± 1.49 ^A,a^
TYR	4	1.14 ± 0.17 ^d^	1.85 ± 0.84 ^B,cd^	2.59 ± 0.65 ^C,c^	4.56 ± 0.74 ^B,b^	7.38 ± 0.63 ^C,a^
	10	1.14 ± 0.17 ^d^	1.28 ± 0.21 ^B,d^	3.34 ± 0.67 ^C,c^	7.95 ± 0.02 ^A,b^	10.72 ± 1.58 ^B,a^
	25	1.14 ± 0.17 ^d^	4.32 ± 0.36 ^A,c^	4.88 ± 0.49 ^B,c^	7.39 ± 0.96 ^A,b^	15.37 ± 2.42 ^A,a^
	30	1.14 ± 0.17 ^c^	2.10 ± 0.39 ^B,c^	6.60 ± 0.58 ^A,b^	7.02 ± 1.32 ^A,b^	15.46 ± 1.51 ^A,a^

ND: not detected. Different capital letters (A–D) indicate significant differences (*p* < 0.05); within each row and for each storage temperature, different lowercase letters (a–e) indicate significant differences (*p* < 0.05). The absence of a letter indicates that no significant differences were found (*p* > 0.05).

**Table 4:** Changes of BAs in canned scallop during storage at different temperatures.

BA Contents/(mg kg^−1^)	Temperature (°C)	Time (Month)				
		0	3	6	9	12
TRM	4	ND ^c^	ND ^c^	1.04 ± 0.19 ^B,b^	1.65 ± 0.38 ^D,a^	1.72 ± 0.32 ^C,a^
	10	ND ^c^	ND ^c^	3.09 ± 0.83 ^B,b^	4.23 ± 0.54 ^C,b^	9.49 ± 1.23 ^B,a^
	25	ND ^d^	ND ^d^	3.14 ± 1.70 ^B,c^	7.02 ± 1.12 ^B,b^	10.42 ± 1.95 ^B,a^
	30	ND ^d^	ND ^d^	5.92 ± 1.41 ^A,c^	9.32 ± 0.92 ^A,b^	13.83 ± 1.21 ^A,a^
PHE	4	0.94 ± 0.06 ^b^	2.49 ± 0.69 ^A,a^	2.52 ± 0.08 ^A,a^	3.21 ± 1.02 ^B,a^	3.23 ± 0.32 ^B,a^
	10	0.94 ± 0.06 ^b^	1.06 ± 0.11 ^B,b^	1.19 ± 0.74 ^C,b^	3.59 ± 0.24 ^B,a^	3.77 ± 0.75 ^B,a^
	25	0.94 ± 0.06 ^d^	2.25 ± 0.20 ^A,c^	1.38 ± 0.03 ^B,d^	5.46 ± 0.39 ^A,b^	6.38 ± 0.92 ^A,a^
	30	0.94 ± 0.06 ^d^	0.41 ± 0.12 ^B,d^	2.04 ± 0.46 ^AB,c^	5.85 ± 0.53 ^A,b^	7.28 ± 0.48 ^A,a^
PUT	4	ND ^c^	ND ^c^	0.79 ± 0.07 ^C,b^	1.32 ± 0.56 ^C,ab^	1.98 ± 1.02 ^C,a^
	10	ND ^d^	ND ^d^	0.58 ± 0.24 ^C,c^	1.43 ± 0.26 ^C,b^	2.32 ± 0.28 ^C,a^
	25	ND ^d^	ND ^d^	1.39 ± 0.03 ^B,c^	3.64 ± 0.19 ^B,b^	5.38 ± 0.37 ^B,a^
	30	ND ^d^	ND ^d^	3.54 ± 0.39 ^A,c^	5.20 ± 0.92 ^A,b^	9.37 ± 0.49 ^A,a^
CAD	4	ND ^e^	0.56 ± 0.13 ^d^	1.04 ± 0.05 ^C,c^	2.99 ± 0.17 ^B,b^	3.31 ± 0.30 ^C,a^
	10	ND ^e^	0.67 ± 0.09 ^d^	1.27 ± 0.16 ^C,c^	3.05 ± 0.21 ^B,b^	3.57 ± 0.25 ^C,a^
	25	ND ^d^	0.77 ± 0.20 ^d^	2.21 ± 0.53 ^B,c^	4.02 ± 0.02 ^A,b^	5.86 ± 1.01 ^B,a^
	30	ND ^c^	0.53 ± 0.18 ^c^	4.02 ± 0.72 ^A,b^	4.09 ± 0.81 ^A,b^	7.92 ± 0.61 ^A,a^
HIS	4	ND ^b^	ND ^C,b^	20.67 ± 2.56 ^C,a^	22.43 ± 2.21 ^C,a^	23.05 ± 0.83 ^C,a^
	10	ND ^c^	12.47 ± 1.31 ^B,b^	34.75 ± 3.21 ^B,a^	37.76 ± 4.18 ^B,a^	39.86 ± 4.12 ^B,a^
	25	ND ^d^	12.68 ± 1.52 ^B,c^	43.75 ± 3.97 ^A,b^	45.29 ± 4.76 ^A,ab^	49.57 ± 2.01 ^A,a^
	30	ND ^d^	27.13 ± 3.22 ^A,c^	38.22 ± 3.05 ^AB,b^	42.69 ± 5.29 ^AB,b^	52.39 ± 3.59 ^A,a^

ND: not detected. Different capital letters (A–D) indicate significant differences (*p* < 0.05); within each row and for each storage temperature, different lowercase letters (a–e) indicate significant differences (*p* < 0.05). The absence of a letter indicates that no significant differences were found (*p* > 0.05).

## Data Availability

Data is contained within the article.

## Supplementary Materials

The following supporting information can be downloaded at: https://www.mdpi.com/article/10.3390/foods11182743/s1, Table S1: Changes of BAs in canned oyster during storage at different temperatures title; Table S2 Chemical Index and Amine Index of the canned seafood samples
