# Supplementary material for: Effect of Storage Temperature and Time on Biogenic Amines in Canned Seafood
Source: Foods. 2022 Sep 7;11(18):2743. doi: 10.3390/foods11182743 (PMC9497643; doi:10.3390/foods11182743)
Supplement: Supplementary file 1 [file foods-11-02743-s001.zip › foods-1882448-supplementary.pdf]

Table S1: Changes of BAs in canned oyster during storage at different temperatures

**Canned oyster** BA/(mg/kg)	**Temperature(°C)**	**Time(month)**				
		**0**	**3**	**6**	**9**	**12**
TRM	4	ND^{c}	ND^{c}	2.48±1.02^{B,b}	9.58±1.99^{B,a}	9.91±1.36^{B,a}
	10	ND^{d}	ND^{d}	2.58±0.49^{B,c}	6.31±0.54^{C,b}	11.74±2.19^{B,a}
	25	ND^{c}	ND^{c}	6.25±2.17^{A,b}	13.97±2.83^{A,a}	15.95±1.46^{A,a}
	30	ND^{d}	ND^{d}	3.56±0.22^{B,c}	13.02±2.64^{AB,b}	17.19±1.29^{A,a}
PHE	4	1.12±0.07^{c}	1.81±0.44^{A,bc}	1.98±0.22^{bc}	2.88±0.25^{C,ab}	3.45±1.23^{B,a}
	10	1.12±0.07^{d}	1.83±0.30^{A,c}	1.95±0.46^{c}	2.54±0.03^{C,b}	4.28±0.21^{B,a}
	25	1.12±0.07^{c}	1.19±0.03^{B,c}	1.59±0.05^{c}	4.58±0.35^{A,b}	7.47±0.72^{A,a}
	30	1.12±0.07^{d}	1.51±0.06^{AB,d}	2.49±0.84^{c}	3.42±0.15^{B,b}	8.47±0.19^{A,a}
PUT	4	ND^{c}	ND^{c}	0.99±0.18^{C,b}	1.23±0.26^{D,b}	2.49±0.08^{D,a}
	10	ND^{d}	ND^{d}	1.57±0.37^{B,c}	2.95±0.43^{C,b}	4.29±0.13^{C,a}
	25	ND^{d}	ND^{d}	1.17±0.05^{BC,c}	5.64±0.21^{B,b}	8.17±0.99^{B,a}
	30	ND^{d}	ND^{d}	4.32±0.40^{A,c}	8.91±1.47^{A,b}	12.47±1.31^{A,a}
CAD	4	ND^{c}	0.41±0.13^{C,bc}	1.03±0.07^{C,b}	2.90±1.05^{C,a}	3.30±0.28^{C,a}
	10	ND^{e}	2.82±0.30^{A,d}	10.38±0.36^{A,c}	15.05±0.39^{A,b}	16.47±1.31^{A,a}
	25	ND^{e}	0.67±0.14^{C,d}	1.32±0.20^{C,c}	3.04±0.29^{C,b}	12.47±0.57^{B,a}
	30	ND^{d}	1.20±0.12^{B,d}	6.36±1.29^{B,c}	10.38±0.93^{B,b}	14.79±1.32^{A,a}
HIS	4	ND^{d}	4.59±2.55^{A,c}	10.65±1.89^{C,b}	12.94±2.27^{B,b}	22.74±2.19^{D,a}
	10	ND^{c}	ND^{B,c}	14.58±2.59^{B,b}	16.33±3.08^{B,b}	28.42±1.55^{C,a}
	25	ND^{d}	ND^{B,d}	18.55±3.01^{AB,c}	29.62±3.46^{A,b}	34.57±1.92^{B,a}
	30	ND^{d}	ND^{B,d}	23.23±3.81^{A,c}	32.06±4.66^{A,b}	44.01±2.74^{A,a}
TYR	4	ND	ND	ND	ND	ND
	10	ND	ND	ND	ND	ND
	25	ND	ND	ND	ND	ND
	30	ND	ND	ND	ND	ND
SPD	4	1.42±0.47^{d}	2.24±0.43^{B,c}	2.07±0.62^{C,cd}	3.04±0.13^{D,b}	3.91±0.21^{D,a}
	10	1.42±0.47^{d}	3.02±0.29^{AB,c}	3.73±0.67^{B,c}	5.95±0.28^{C,b}	6.19±0.14^{C,a}
	25	1.42±0.47^{d}	3.91±1.03^{A,c}	4.99±0.07^{B,c}	9.25±1.32^{B,b}	12.57±1.29^{B,a}
	30	1.42±0.47^{c}	2.07±0.12^{B,c}	6.94±1.17^{A,b}	14.23±2.05^{A,a}	15.38±0.58^{A,a}
SPM	4	ND^{d}	ND^{d}	3.59±0.36^{B,c}	4.95±0.93^{C,b}	6.31±0.64^{B,a}
	10	ND^{c}	ND^{c}	3.58±0.46^{B,b}	6.02±1.09^{BC,a}	6.53±0.81^{B,a}
	25	ND^{d}	ND^{d}	5.81±0.11^{A,c}	7.52±0.63^{AB,b}	9.75±0.19^{A,a}
	30	ND^{d}	ND^{d}	3.83±0.35^{B,c}	8.61±0.79^{A,b}	10.31±1.24^{A,a}

ND: not detected. Within each column and for each storage time of each amine, different capital letters (A–D) indicate significant differences ($p < 0.05$); within each row and for each storage temperature, different lowercase letters (a–e) indicate significant differences ($p < 0.05$). The absence of a letter indicates that no significant differences were found ($p > 0.05$).

Table S2: Chemical Index and Amine Index of the canned seafood samples

Sample	Storage temperature	Storage time	Chemical Index	Amine Index	Classification
Canned mud carp	4	0	0.41	18.67	Good quality
		3	1.53	39.38	Good quality
		6	2.14	37.03	Good quality
		9	2.13	52.21	Good quality
		12	2.29	53.28	Good quality
	10	0	0.41	18.67	Good quality
		3	0.82	36.31	Good quality
		6	2.11	57.66	Good quality
		9	2.09	55.16	Good quality
		12	2.19	53.63	Good quality
	25	0	0.41	18.67	Good quality
		3	3.88	64.76	Poor quality
		6	2.09	53.65	Good quality
		9	1.46	46.69	Good quality
		12	1.26	43.40	Good quality
	30	0	0.41	18.67	Good quality
		3	3.15	49.59	Poor quality
		6	1.89	50.63	Good quality
		9	2.32	54.78	Good quality
		12	1.93	50.97	Good quality
Canned sardine	4	0	0.36	13.69	Good quality
		3	1.33	38.98	Good quality
		6	1.39	44.56	Good quality
		9	1.11	42.53	Good quality
		12	1.02	39.65	Good quality
	10	0	0.36	13.69	Good quality
		3	1.95	47.55	Good quality
		6	1.69	48.33	Good quality
		9	1.66	45.48	Good quality
		12	1.50	43.22	Good quality
	25	0	0.36	13.69	Good quality
		3	0.25	9.91	Good quality
		6	2.02	52.78	Good quality
		9	2.20	49.74	Good quality
		12	2.02	46.57	Good quality
	30	0	0.36	13.69	Good quality
		3	2.62	60.10	Poor quality
		6	1.46	47.18	Good quality
		9	1.56	47.51	Good quality
		12	1.59	42.39	Good quality
Canned mantis shrimp	4	0	5.61	65.31	Good quality
		3	9.27	69.23	Good quality
		6	17.50	74.90	Good quality
		9	25.10	74.61	Good quality
		12	28.57	68.45	Good quality
	10	0	5.61	65.31	Good quality
		3	18.01	74.85	Good quality
		6	28.02	76.20	Poor quality
		9	41.54	70.97	Good quality
		12	50.93	70.28	Good quality

	25	0	5.61	65.31	Good quality
		3	17.99	70.88	Good quality
		6	33.69	75.57	Poor quality
		9	52.00	72.87	Good quality
		12	73.52	69.94	Good quality
	30	0	5.61	65.31	Good quality
		3	12.22	58.44	Good quality
		6	54.63	77.18	Poor quality
		9	73.04	77.88	Poor quality
		12	95.93	73.46	Poor quality
Canned scallop	4	0	0.00	0.00	Good quality
		3	0.56	18.36	Good quality
		6	22.50	86.34	Good quality
		9	26.74	84.62	Good quality
		12	28.34	85.13	Good quality
	10	0	0.00	0.00	Good quality
		3	13.14	92.53	Poor quality
		6	36.60	89.53	Good quality
		9	42.24	84.38	Good quality
		12	45.75	77.53	Good quality
	25	0	0.00	0.00	Good quality
		3	13.45	85.67	Good quality
		6	47.35	91.29	Poor quality
		9	52.95	80.93	Good quality
		12	60.81	78.35	Poor quality
	30	0	0.00	0.00	Good quality
		3	27.66	98.54	Poor quality
		6	45.78	85.19	Good quality
		9	51.98	77.41	Good quality
		12	69.68	76.75	Poor quality
canned oyster	4	0	0.00	0.00	Good quality
		3	1.54	55.25	Good quality
		6	1.90	55.60	Good quality
		9	1.90	45.50	Good quality
		12	2.54	54.75	Good quality
	10	0	0.00	0.00	Good quality
		3	0.70	36.77	Good quality
		6	3.19	70.99	Good quality
		9	2.65	62.25	Good quality
		12	3.58	63.12	Good quality
	25	0	0.00	0.00	Good quality
		3	0.14	11.61	Good quality
		6	1.78	53.02	Good quality
		9	2.15	52.02	Good quality
		12	2.37	54.69	Good quality
	30	0	0.00	0.00	Good quality
		3	0.39	25.10	Good quality
		6	2.88	66.84	Good quality
		9	2.15	56.66	Good quality
		12	2.67	58.12	Good quality

Samples description, including time of storage, are detailed in subsection 2.3 of Materials and Methods, RSD<5%.
